# Electroconvulsive therapy efficacy in adolescents with mental illness: A retrospective comparison

**DOI:** 10.3389/fpsyt.2022.990660

**Published:** 2022-09-09

**Authors:** Qi Si, Xinyue Zhang, Jiaxi Lei, Congxin Chen, Fangfang Ren, Guoxin Xu, Yuan Li, Yuxiu Sui

**Affiliations:** ^1^Department of Psychiatry, The Affiliated Brain Hospital of Nanjing Medical University, Nanjing Brain Hospital, Nanjing, China; ^2^Department of Psychiatry, Shenzhen Kangning Hospital, Shenzhen, China; ^3^Department of Psychiatry, The Fourth People's Hospital of Chengdu, Chengdu, China

**Keywords:** adolescent, mental illness, electroconvulsive therapy, retrospective study, efficacy

## Abstract

**Background:**

There is limited evidence on the efficacy of electroconvulsive therapy (ECT) in adolescents with mental illness. The present study reported outcomes of adolescents with mental illness treated with ECT aimed at providing evidence for large-scale feasibility.

**Objectives:**

The primary objective of this trial was to examine the differences in demographic and clinical data between responders and non-responders. The secondary objective was to determine whether ECT produced differential readmission rates, the burden of oral medication, and social function in responders and non-responders in the long term.

**Methods:**

Patients aged 14–18 years diagnosed with schizophrenia (SCZ), major depressive disorder (MDD), or bipolar disorder (BD) who received ECT between 2015 and 2020 were included in the study. Demographic and clinical data were compared, and both short-term and long-term outcomes were assessed: response on the Clinical Global Impressions-Improvement scale and readmission at follow-up. The independent-sample *t*–test was used to compare the continuous variables and the *X*^2^ test was used to compare the dichotomous variables with statistical significance at *P* ≤ 0.05.

**Results:**

Four hundred ten adolescents (aged 14–18 years, 53.90% female) received ECT for SCZ, MDD, and BD. The response rate for SCZ, MDD, and BD were 65.61, 78.57, and 69.95%, respectively. Both SCZ (*P* = 0.008) and BD (*P* = 0.008) groups had a significant elder age in responders than in non-responders. Besides that MDD responders had a significantly larger number of ECT sessions than non-responders (*P* = 0.046), the study failed to find a significant difference in other ECT parameters. A significantly higher proportion of readmission was found in BD non-responders than in responders (*P* = 0.029), there was no difference in the rate of readmission in other diagnostic groups.

**Conclusions:**

These data suggested that ECT is an effective treatment for adolescents with severe mental illness, and the rate of readmission was low in the long term. The present study supports that large-scale systematic studies are warranted for further investigation of the response rate of ECT for treating adolescents with mental illness.

## Introduction

Electroconvulsive therapy (ECT) is a suitable treatment for adults with severe mental disorders. In Asian and African countries, ECT is mainly used for psychotic disorders, in patients with schizophrenia (SCZ) and other psychotic conditions (1). ECT for SCZ has increased over the past years, a national survey demonstrated that ECT treatment of SCZ increased from 4.7 to 7.7% between 2006 and 2012 in China (2). A large body of research in Taiwan showed that ECT could treat SCZ effectively and significantly decrease the rate of re-hospitalization, correspondingly, the total medical expenses increased significantly in the non-ECT patients, but not in the ECT patients (3). ECT also has a positive effect on the clinical response of adults with treatment-resistant SCZ (4). A trial determined the rates of ECT used in adolescents with catatonia in schizophrenia spectrum disorders in America, the results suggested that ECT was used for only 13% of adolescents with catatonia when comorbid schizophrenia spectrum disorders, and a high rate of ECT use was evident for Whites compared with the other race and also was seen in private health insurance beneficiaries (5). These characteristics in the utilization of ECT in adolescents were also found by Trivedi et al. (6). In the United States and European countries, ECT was predominantly used in patients with affective disorders, treated severe depression effectively, and could reduce the risk of suicide (7, 8). A recent meta-analysis demonstrates that the response rates for adults major depressive disorder (MDD) range from 58 to 70% (9). A retrospective cohort study showed the risk of suicide was found to be significantly reduced for adults with MDD who received ECT (10). ECT was also effective for bipolar disorder (BD), a previous study showed that the response rates for bipolar depression, mixed-state, and mania treated by ECT were 68.1, 72.9, and 75%, respectively (11). ECT is relatively well tolerated in adults, with typical side effects being transient cognitive problems, headache, nausea, and muscle soreness (12).

SCZ, BD, MDD, and other severe mental illnesses are also common conditions in adolescence that can impair the social, academic, or daily functioning of affected individuals, quite often predisposing them to a high risk of self-injury or suicide. Rates of self-injury are substantially higher amongst adolescents than in adults, ranging from 7.5–46.5% in adolescents and 4–23% in adults (13). However, there is a lack of available and effective interventions for adolescents in the acute phase of mental illness or for those with tendencies toward suicide and self-injury. Previous literature showed that the first-line antidepressants may not achieve satisfactory effect in 60% of adolescents with MDD (14). Some limitations associated with interventions for acute onset of adolescents with mental disorders include slow effectiveness, lack of well-tolerated and effective pharmaceuticals, and limited pharmaceutical therapeutic options (15, 16). Besides that, there are hidden safety risks in antidepressant medication, such as selective serotonin reuptake inhibitors (SSRIs) could increase the risk of suicidality and self-injury in youth (17, 18). Therefore, ECT could be regarded as an effective treatment.

Although ECT is considered an effective and safe treatment for adults; however, the use of ECT is much less common in adolescents than in adults, for multiple reasons (19). There is a lack of studies regarding the efficacy of ECT in adolescents or children. Due to ethical review, the large sample, randomized control study could not carry out. A previous study estimated the knowledge and attitudes toward the use of ECT in children and adolescents, among child and adolescent psychiatrists, the results showed that about half of the respondents possessed minimal knowledge about the use of ECT in children and adolescents, three-quarters of respondents lack confidence for the efficacy of ECT in children and adolescents, and the majority of respondents regarded ECT as treatment of last resort (20). Similar results were found in the other two studies (21, 22). Moreover, there is specific legislation that inhibits the use of ECT for children under certain ages in some countries and areas, which also limits the research (23). For the above reasons, the proportion of ECT used in adolescents with mental illness only accounts for 1%, which is much lower than in adults (19).

In recent years, the literature supporting the use of ECT in adolescents is growing. A recent study (835 samples) reviewed adolescents with schizophrenia from 2007 to 2016 in a single Chinese academic medical center. This study found that the frequency of ECT use was 49.2% and ECT use was independently and positively associated with gender, and high risk for suicide (24). A retrospective study examined the association between baseline futures and clinical outcomes, the results suggested that ECT is safe and efficacy, but the clinical response was not predicted by demographic data (23). However, there are also some limitations in those studies, such as the lack of ECT parameters, long-term social function outcomes, and the rate of readmission. Herein, we compared the demographic and clinical data of adolescents treated with ECT at a single medical center in the People's Republic of China. The objectives of our study were to examine (1) the differences in baseline data between ECT responders and non-responders (2) the future of ECT parameters in ECT responders or non-responders (3) the differences in the rate of readmission, social function, and burden of oral medication between patients with and without response in long-term after ECT treatment.

## Methods

### Data source

The protocol for the study was approved by the institutional review board at the Affiliated Brain Hospital of Nanjing Medical University. We reviewed the electronic medical records and the electronic prescribing system of SCZ, BD, and MDD patients aged 14–18 years old who received ECT for the first time at the Affiliated Brain Hospital of Nanjing Medical University, from January 2015 through December 2020. Clinical diagnoses of SCZ, MDD, and BD were based on the International Classification of Diseases, Tenth Edition (ICD-10). The exclusion criteria were as follows: diagnosis of other mental disorders; drug or alcohol abuse or dependence in the last 6 months; ECT in the past. Diagnoses at admission, diagnosis changes if patients readmission at follow-up, oral medication, and ECT parameters were collected. We collected demographic data including sex, age, family history of psychiatric disorders, first episode age, duration of illness, and duration of hospitalization.

Adolescents who received ECT were evaluated by at least 2 board-certified psychiatrists, and the medical director of the hospital approved ECT according to the hospital policy. Patients and their legal guardians were provided with information about the diagnosis, treatment options, and the risks and benefits of ECT. Written informed consent from the patients' guardians was obtained prior to starting ECT.

We recorded the number of ECT sessions and collected ECT parameters during the implementation of ECT, including seizure duration time (SDT), seizure energy index, and positive suppression index (PSI). For patients who received more than one ECT treatment series during their adolescence, we analyzed data on the first ECT series only. The course of ECT generally comprised 6–12 sessions for each patient, 3 times per week, between 9 a.m. and 11 a.m. Electroconvulsive therapy was administered using a Thymatron DGx device. Anesthesia was induced with propofol (1–1.5 mg/kg) accompanied by succinylcholine (0.5 mg/kg) and oxygenation. Patients were pre-oxygenated and then manually ventilated using a valve mask and 100% oxygen when adequate muscle relaxation was achieved. A brief pulse wave device with bitemporal electrode placement was used. All patients received hyperventilation before the stimulus was administered.

### Outcomes measures

All patients were assigned a Global Clinical Impressions–Improvement scale (CGI-I) score (25). The score of 1 indicated “very much improved” and 2 indicated “much improved” on the CGI-I. All outpatients were assigned a Personal and Social Performance scale (PSP) score (26). All assessments were determined by independent reviews made by 2 primary physicians. If there was a disagreement between 2 physicians, they reviewed the medical record together, thus reaching a consensus.

### Statistical analyses

The Statistical Product and Service Solutions (SPSS) version 24.0 was used to analyze the data. Baseline variables included sex (dichotomous), age (continuous), family history of psychiatric disorders (dichotomous), the first episode age (continuous), illness duration (continuous), and days in the hospital (continuous). ECT numbers (continuous), SDT (continuous), SEI (continuous), and PSI (continuous) were also measured. The medication received while on ECT course was also recorded. For outpatients, the types of oral medication were further distinguished, regardless if they were increased or not. To compare the differences in continuous variables between responsive and non-responsive patients and those who were readmitted and not readmitted, the independent-sample *t*-test was used, and the *X*^2^ test was used to compare the dichotomous variables. Statistical significance was set at *P* < 0.05 (bilateral).

## Results

### Demographic characteristics and clinical data

A total of 410 adolescents received ECT for mental disorders at the Affiliated Brain Hospital of Nanjing Medical University. 53.90% patients were female, and the mean (SD) age at the start of the treatment series was 16.67 (1.10) years. There were 28.78% patients with a family history of psychiatric disorders. The mean (SD) age at the first episode was 15.07 (1.69) years, and the age range was 10~18 years. The duration of illness ranged from 2 weeks−10 years, and the mean (SD) duration of illness was 22.59 (19.66) months. Patients required a mean (SD) of 34.60 (18.28) days in the hospital. Among patients who were readmitted, the diagnosis was changed in 6 patients. The diagnosis of 1 MDD patient and 1 BD patient were changed to SCZ; the diagnosis of 3 MDD patients and 1 SCZ patient were changed to BD.

#### SCZ patients

For 157 SCZ patients, the response rate was 65.61%. Among 103 patients who responded to ECT, 30.10% patients were female; the mean (SD) age was 16.87 (1.09) years; 29.13% patients with a family history of psychiatric disorders; the mean (SD) age at the first episode was 15.30 (1.84) years; the mean (SD) duration of illness was 21.41 (19.94) months; the mean (SD) duration of hospitalization was 39.71 (19.47) days. Among 54 non-responders to ECT, 46.30% patients were female; the mean (SD) age was 16.39 (1.02) years; 20.37% patients were with a family history of psychiatric disorders; the mean (SD) age at the first episode was 15.19 (1.55) years; the mean (SD) duration of illness was 18.21 (17.57) months; the mean (SD) duration of hospitalization was 39.63 (32.73) days. There was a significant difference in age between responders and non-responders (*t* = −2.71, *P* = 0.008) (Table 1).

**Table 1:** Demographic and clinical characteristics of 157 SCZ patients. Comparison between patients with and without response after ECT course.

**Dichotomous variables^a^**	**Responders (*n* = 103)**	**Non-responders (*n* = 54)**	***X^2^***	***P* ^c^**
Female	31 (30.10%)	25 (46.30%)	0.77	0.381
Family history of psychiatric disorders	30 (29.13%)	11 (20.37%)	0.11	0.744
The kinds of oral medication increased	55 (53.40%)	23 (42.59%)	1.65	0.198
Readmission	21 (20.39%)	7 (12.96%)	1.33	0.248
Antipsychotics				
Olanzapine	43 (41.75%)	18 (33.33%)	N/A	N/A
Quetiapine	5 (4.85%)	0 (0.00%)	N/A	N/A
Clozapine	18 (17.48%)	13 (24.07%)	N/A	N/A
Risperidone	30 (29.13%)	18 (33.33%)	N/A	N/A
Paliperidone	9 (8.74%)	3 (5.56%)	N/A	N/A
Perosprione	1 (0.97%)	1 (1.85%)	N/A	N/A
Amisulpride	28 (27.18%)	10 (18.52%)	N/A	N/A
Aripiprazole	20 (19.42%)	13 (24.07%)	N/A	N/A
Haloperidol	0 (0.00%)	2 (3.70%)	N/A	N/A
Perphenazine	3 (2.91%)	1 (1.85%)	N/A	N/A
**Continuous variables** ^b^	**Responders (*****n*** = **103)**	**Non-responders (*****n*** = **54)**	***t***	*P* ^d^
Age (years)	16.87 (1.09)	16.39 (1.02)	−2.71	0.008^*^
First episode age (years)	15.30 (1.84)	15.19 (1.55)	−0.39	0.694
Duration of illness (months)	21.41 (19.94)	18.21 (17.57)	−1.04	0.302
Duration of hospitalization (days)	39.71 (19.47)	39.63 (32.73)	−0.02	0.985
PSP scores	74.03 (5.91)	74.83 (4.87)	0.86	0.392
ECT parameters				
Number of ECT sessions	7.67 (1.90)	7.76 (1.92)	0.28	0.781
SDT (seconds)	38.73 (10.09)	36.82 (9.88)	−1.14	0.257
SEI	51693.35 (42173.21)	38505.52 (36082.34)	−1.95	0.053
PSI	81.65 (12.29)	80.87 (8.95)	−0.41	0.680

a n (%).

b mean (SD).

c Chi-square test.

d independent-sample t–test.

SCZ, schizophrenia; ECT, electroconvulsive therapy; PSP, Person and Social Performance scale; SDT, seizure duration time; SEI, seizure energy index; PSI, postictal suppression index.

* P < 0.050.

For antipsychotics that responders received, olanzapine (41.75%) was the most common antipsychotic prescribed to patients undergoing ECT, followed by risperidone (29.13%), amisulpride (27.18%), aripiprazole (19.42%), clozapine (17.48%), paliperidone (8.74%), quetiapine (4.85%), and perphenazine (2.91%). One patient was treated with perosprione. For non-responders, olanzapine (33.33%) and risperidone (33.33%) were both the most common antipsychotic, followed by clozapine (24.07%) and aripiprazole (24.07%), amisulpride (18.52%), paliperidone (5.56%), and haloperidol (3.70%). Two patients were respectively treated with perosprione and perphenazine (Table 1).

#### MDD patients

For 70 MDD patients, the response rate was 78.57%. Among 55 who responded to ECT, 61.82% patients were female; the mean (SD) age was 16.60 (1.10) years; 34.55% patients with a family history of psychiatric disorders; the mean (SD) age at the first episode was 14.78 (1.59) years; the mean (SD) duration of illness was 25.00 (18.71) months; the mean (SD) duration of hospitalization was 31.04 (10.06) days. Among 15 non-responders to ECT, 60.00% patients were female; the mean (SD) age was 16.33 (1.11) years; 20.00% patients with a family history of psychiatric disorders; the mean (SD) age at the first episode was 15.20 (1.57) years; the mean (SD) duration of illness was 16.72 (17.56) months; the mean (SD) duration of hospitalization was 26.40 (10.03) days. There was no significant difference between responders and non-responders (Table 2).

**Table 2:** Demographic and clinical characteristics of 70 MDD patients. Comparison between patients with and without response after ECT course.

**Dichotomous variables^a^**	**Responders (*n* = 55)**	**Non–responders (*n* = 15)**	***X^2^***	***P* ^c^**
Female	34 (61.82%)	9 (60.00%)	0.02	0.898
Family history of psychiatric disorders	19 (34.55%)	3 (20.00%)	1.16	0.282
The kinds of oral medication increased	30 (54.55%)	4 (26.67%)	3.67	0.055
Readmission	9 (16.36%)	3 (20.00%)	0.11	0.740
Antidepressants				
Fluoxetine	6 (10.91%)	5 (33.33%)	N/A	N/A
Paroxetine	1 (1.82%)	0 (0.00%)	N/A	N/A
Sertraline	31 (56.36%)	4 (26.67%)	N/A	N/A
Fluvoxamine	2 (3.64%)	1 (6.67%)	N/A	N/A
Escitalopram	11 (20.00%)	3 (20.00%)	N/A	N/A
Venlafaxine	4 (7.27%)	2 (13.33%)	N/A	N/A
Milnacipran	1 (1.82%)	0 (0.00%)	N/A	N/A
Mirtazapine	1 (1.82%)	2 (13.33%)	N/A	N/A
Agomelatine	4 (7.27%)	0 (0.00%)	N/A	N/A
MSs				
Lithium	5 (9.09%)	0 (0.00%)	N/A	N/A
Valproate	2 (3.64%)	3 (20.00%)	N/A	N/A
**Continuous variables** ^b^	**Responders (*****n*** = **55)**	**Non–responders (*****n*** = **15)**	***t***	*P* ^d^
Age (years)	16.60 (1.10)	16.33 (1.11)	−0.83	0.409
First episode age (years)	14.87 (1.59)	15.20 (1.57)	−0.71	0.480
Duration of illness (months)	25.00 (18.71)	16.72 (17.56)	−1.54	0.129
Duration of hospitalization (days)	31.04 (10.06)	26.40 (10.03)	−1.58	0.118
PSP scores	78.62 (5.23)	80.40 (5.58)	1.15	0.253
ECT parameters				
Number of ECT sessions	7.44 (1.34)	6.60 (1.64)	−2.04	0.046^*^
SDT (seconds)	41.15 (14.89)	47.71 (19.26)	1.42	0.161
SEI	55631.31 (35766.59)	52116.78 (80975.57)	−0.25	0.805
PSI	80.01 (12.43)	80.71 (10.18)	0.23	0.824

a n (%).

b mean (SD).

c Chi–square test.

d independent–sample t test.

MDD, major depressive disorder; ECT, electroconvulsive therapy; MSs, Mood stabilizers; PSP, Person and Social Performance scale; SDT, seizure duration time; SEI, seizure energy index; PSI, postictal suppression index.

* P < 0.050.

The most common antidepressant which responders received was sertraline (56.36%), followed by escitalopram (20.00%), fluoxetine (10.91%), venlafaxine (7.27%) and agomelatine (7.27%), and fluvoxamine (3.64%). Three patients were treated with paroxetine, milnacipran, and mirtazapine, respectively. Five patients combined with lithium, and two with valproate. For non-responders, the most common antidepressant was fluoxetine (33.33%), followed by sertraline (26.67%), escitalopram (20.00%), venlafaxine (13.33%) and mirtazapine (13.33%). One patient was treated with fluvoxamine. Three patients combined with valproate (Table 2).

#### BD patients

For 183 BD patients, the response rate was 69.95%. Among 128 responders of ECT, 63.28% patients were female; the mean (SD) age was 16.83 (1.04) years; 27.34% patients with a family history of psychiatric disorders; the mean (SD) age at the first episode was 15.05 (1.68) years; the mean (SD) duration of illness was 25.10 (21.03) months; the mean (SD) duration of hospitalization was 31.31 (11.82) day; the most common diagnosis was bipolar depressive episode (59.38%), followed by mania episode (26.56%), mixed episode (12.50%), and rapid cycling (1.56%). Among 55 non-responders of ECT, 74.55% patients were female; the mean (SD) age was 16.36 (1.16) years; 20 patients (36.36%) with a family history of psychiatric disorders; the mean (SD) age at the first episode was 14.75 (1.68) years; the mean (SD) duration of illness was 22.47 (18.78) months; the mean (SD) duration of hospitalization was 33.55 (13.18) day; the most common diagnosis was bipolar depressive episode (67.27%), followed by mania episode (20.00%), mixed episode (12.73%). There was a significant difference in age between responders and non-responders (*t* = −2.68, *P* = 0.008) (Table 3).

**Table 3:** Demographic and clinical characteristics of 183 BD patients. Comparison between patients with and without response after ECT course.

**Dichotomous variables^a^**	**Responders (*n* = 128)**	**Non–responders (*n* = 55)**	***X^2^***	***P* ^c^**
Female	81 (63.28%)	41 (74.55%)	2.20	0.138
Family history of psychiatric disorders	35 (27.34%)	20 (36.36%)	1.49	0.222
The kinds of oral medication increased	64 (50.00%)	26 (47.27%)	0.11	0.735
Diagnosis				
Bipolar depressive episode, without psychosis	40 (31.25%)	25 (45.45%)	N/A	N/A
Bipolar depressive episode, with psychosis	36 (28.13%)	12 (21.82%)	N/A	N/A
Bipolar mania episode, without psychosis	21 (16.41%)	8 (14.55%)	N/A	N/A
Bipolar mania episode, with psychosis	13 (10.16%)	3 (2.34%)	N/A	N/A
Bipolar mixed episode	16 (12.50%)	7 (12.73%)	N/A	N/A
Bipolar rapid cycling	2 (1.56%)	0 (0.00%)	N/A	N/A
Readmission	25 (19.53%)	19 (34.55%)	4.75	0.029^*^
MSs				
Lithium	96 (75.00%)	36 (65.45%)	N/A	N/A
Valproate	46 (35.94%)	22 (40.00%)	N/A	N/A
Lamotrigine	3 (2.34%)	2 (3.64%)	N/A	N/A
SGAPs				
Olanzapine	39 (30.47%)	22 (40.00%)	N/A	N/A
Quetiapine	72 (56.25%)	28 (50.91%)	N/A	N/A
Risperidone	9 (7.03%)	2 (3.64%)	N/A	N/A
Paliperidone	2 (1.56%)	0 (0.00%)	N/A	N/A
Aripiprazole	14 (10.94%)	7 (12.73%)	N/A	N/A
**Continuous variables** ^b^	**Responders (*****n*** = **128)**	**Non–responders (*****n*** = **55)**	***t***	*P* ^d^
Age (years)	16.83 (1.04)	16.36 (1.16)	−2.68	0.008*
First episode age (years)	15.05 (1.68)	14.75 (1.68)	−1.14	0.255
Duration of illness (months)	25.10 (21.03)	22.47 (18.78)	−0.80	0.423
Duration of hospitalization (days)	31.31 (11.82)	33.55 (13.18)	1.08	0.282
PSP scores	77.16 (4.99)	76.89 (3.60)	−0.42	0.678
ECT parameters				
Number of ECT sessions	6.91 (1.50)	6.76 (1.41)	−0.63	0.527
SDT (seconds)	41.22 (16.97)	40.94 (11.82)	−0.11	0.911
SEI	52274.47 (41636.08)	50248.92 (47825.98)	−0.29	0.773
PSI	80.33 (12.44)	79.39 (12.20)	−0.47	0.640

a n (%).

b mean (SD).

c Chi–square test.

d independent–sample t test.

BD, bipolar disorder; ECT, electroconvulsive therapy; MSs, Mood stabilizers; SGAPs, second–generation antipsychotics; PSP, Person and Social Performance scale; SDT, seizure duration time; SEI, seizure energy index; PSI, postictal suppression index.

* P < 0.050.

For responders, the most common mood stabilizer (MS) was lithium (75.00%), followed by valproate (35.94%), and lamotrigine (2.34%); the most common second-generation antipsychotic (SGAP) was quetiapine (56.25%), followed by olanzapine (30.47%), aripiprazole (10.94%), risperidone (7.03%), and paliperidone (1.56%). For non-responders, the most common MS was lithium (65.45%), followed by valproate (40.00%), and lamotrigine (3.64%); the most common SGAP was quetiapine (50.91%), followed by olanzapine (40.00%), aripiprazole (12.73%), and risperidone (3.64%) (Table 3).

### ECT characteristics

#### SCZ patients

For responders, the mean (SD) number of ECT sessions was 7.67 (1.90); the mean (SD) SDT was 38.73 (10.09) seconds; the mean (SD) SEI was 51693.35 (42173.21), the mean (SD) PSI was 81.65 (12.29). For non-Responders, the mean (SD) number of ECT sessions was 7.76 (1.92); the mean (SD) SDT was 36.82 (9.88) seconds; the mean (SD) SEI was 38505.52 (36082.34), the mean (SD) PSI was 80.87 (8.95). While the study failed to show a significant difference between responders and non-responders for these outcomes specifically, numerical advantage of SEI favoring the responders was observed (Table 1).

#### MDD patients

For responders, the mean (SD) number of ECT sessions was 7.44 (1.34); the mean (SD) SDT was 41.15 (14.89) sec; the mean (SD) SEI was 55631.31 (35766.59), the mean (SD) PSI was 80.01 (12.43). For non-responders, the mean (SD) number of ECT sessions was 6.60 (1.64); the mean (SD) SDT was 47.71 (19.26) sec; the mean (SD) SEI was 52116.78 (80975.57), the mean (SD) PSI was 80.71 (10.18). There was no significant difference in SDT, SEI and PSI for two groups. But the significant larger number of ECT sessions were received for responders than for non-responders (*t* = −2.04, *P* = 0.046) (Table 2).

#### BD patients

For responders, the mean (SD) number of ECT sessions was 6.91 (1.50); the mean (SD) SDT was 41.22 (16.97) sec; the mean (SD) SEI was 52274.47 (41636.08), the mean (SD) PSI was 80.33 (12.44). For non-responders, the mean (SD) number of ECT sessions was 6.76 (1.41); the mean (SD) SDT was 40.94 (11.82) sec; the mean (SD) SEI was 50248.92 (47825.98), the mean (SD) PSI was 79.39 (12.20). No significant was found in ECT parameters between two groups (Table 3).

### Follow-up outcomes

At the end of the follow-up, the readmission rate was 20.49%. There was found a significant higher readmission rate in BD non-responders than in BD responders (34.55% *v*. 19.53%, *X*^2^ = 4.75, *P* = 0.029) (Table 3). For the other two diagnostic groups, no significant difference was found in readmission rate between responders and non-responders. For outpatients, the mean (SD) PSP scores was 76.35 (5.40), and no significant difference was found respectively in three diagnostic groups between responders and non-responders. While patients who were responded for ECT treatment in three diagnostic groups all had a high proportion of increasing the kinds of oral medication at outpatient setting, no significant difference was found between responders and non-responders at follow-up.

## Discussion

In this study, we reviewed the demographic and clinical data of adolescents with certain severe mental disorders treated with ECT at a signal academic medical center. Our research provided evidence to support the efficacy of ECT in adolescents, and also demonstrated the long-term outcome. Our results showed the highest response rate was observed in MDD (78.57%), followed by BD (69.95%), and the lowest response rate was SCZ (65.61%). For SCZ and BD patients, we found that there was a significant difference in age between responders and non-responders. The largest number of ECT sessions was observed in SCZ, which was significantly more than in BD and MDD. At follow-up, we found significant differences in the first episode age, duration of illness, duration of hospitalization, and SEI between patients with or without readmission. The PSP scores showed that the social function recovered well for all three diagnosis groups.

The samples in this study had severe mental illness, but the improvement was significantly well after a course of ECT. The overall response rate in our samples was 69.76%, which is lower than reported in other recent retrospective studies. Two retrospective studies (107 samples and 51 samples, respectively) reviewed the outcomes of adolescents treated with ECT in America, both reporting response rates of 77% (23, 27). In Asia, a cohort study (22 samples) from multi-sites reported a response rate of 77% (28). Our findings revealed a lower response rate compared to those studies, which might be due to a greater proportion of patients with SCZ in the present study compared to American and Indian samples. The present study included 157 SCZ patients (38.29%), which is much higher than the 4.67% of patients with SCZ included in America and 17.65% in India. Moreover, the sample sizes of previous studies were smaller compared to the present study, and there were differences in race and region. Consequently, more large samples, multi-site, and prospective studies are needed to evaluate this issue further.

In our study, although the efficacy of ECT in the treatment of SCZ was lower than affective disorders, the results showed that the majority of adolescents with SCZ had experienced clinically significant improvement. Wang et al. reviewed adolescents with SCZ treated by ECT in China (326 samples), and their results suggested that the response rate was 65%, which was very close to our results (24). A retrospective chart review evaluated the clinical profile of adolescents who had received ECT, the results showed that the rate of remission for SCZ was lower than MDD and mania, which was similar to our results (29). Previous studies have shown that SCZ affects neurodevelopment during the period of adolescence or earlier, the treatment is challenging (30). A meta-analysis in over 18,000 subjects reported that brain loss in SCZ was related to a combination of neurodevelopmental processes reflected in intracranial volume reduction (31). Duan et al. compared the hippocampus volume between patients with adolescent-onset SCZ (AOS) (36 samples) and typically developing controls (TDC) (30 samples), providing evidence on hippocampus volume reduction in AOS, acting as a mediator between hippocampal morphometry and negative symptom (32). The early onset adolescents often have negative symptoms and cognitive deficits, which were predicted poor function outcomes (33). ECT had poor efficacy among early-onset adolescents with negative symptoms, especially first-episode SCZ. In summary, clinicians should pay more attention to the assessment of early-onset SCZ adolescents and formulate a personalized treatment.

For MDD, the response rate was higher than other diagnoses, and the majority were in stable condition at follow-up. Castaneda-Ramirez et al. reviewed 41 studies (601 samples) on the use of ECT for treatment-resistant mood disorders in children and adolescents, and they reported the response rate of ECT ranging from 53–77%, which was consistent with our results (34). ECT could relieve severe depressive symptoms, such as self-injury or suicide, in the short term. A retrospective case series of ECT used in MDD and anorexia nervosa (AN) showed that ECT is safe and well-tolerated in AN with severe comorbid treatment-resistant MDD and/or with increased suicide risk (35). A retrospective chart review found that ECT could decrease suicidal behavior, reduce depressive symptoms, and improve overall function at follow-up after 1 year (36). But fewer literature reported the readmission rate in adolescents with MDD treated by ECT, our study provided evidence for this. The results of the study showed that ECT is a safe and effective treatment for adolescents with MDD.

According to our findings, ECT resulted in an effective treatment for adolescents with BD, the rate of readmission was higher than MDD and SCZ. Pierson et al. reviewed the outcomes of manic patients treated by ECT (16 samples), reporting a response rate of 88% (23). In the other retrospective study (33 samples) of BD patients, the response rate was 93% (37). Medda et al. evaluated the long-term outcome of patients with bipolar depression or mixed state, responsive to ECT (70 samples), they found that 33% of patients showed a depressive relapse and 10% a mixed relapse at the end of the follow-up (38). A meta-analysis reviewed 8 studies on recurrence after the first episode of mania, revealing the recurrence rate of 25.7% at 6 months and 41.0% at 1 year, where a greater risk of recurrence was associated with younger age at onset (39). Based on our outcomes, the readmission rate was high at the follow-up that suggesting a high risk of relapse in the long term, which is consistent with the previous literature on adult patients. For adolescents with BD who received ECT, there is limited evidence of relapse rate. However, ECT is an effective treatment for BD, the factors that influence the rate of relapse after the ECT needs to be further examined.

Our results showed that the low SEI and increase in oral medication were associated with a high risk of readmission. SEI and other parameters have been reported as related to the effectiveness of ECT. They can also be used to assess seizure quality (40). The results suggested that the seizure quality influenced the clinical outcome in the long term. The association between oral medication and readmission suggested that adolescents who could not tolerate medication treatment or lacked treatment compliance had a high risk of relapse. To the best of our knowledge, there are no previous studies on seizure quality and medication treatment that could influence clinical outcomes for adolescents in the long term. Accordingly, this issue should be further examined by future studies.

The PSP score showed that the social function of adolescents recovered well after the ECT course. There have been few studies on the improvement of the social function of adolescents after ECT in long term. A previous study reviewed the long-term improvement of the overall function of adolescents with mood disorders after ECT, the results showed that the score of the Mini-Mental State Examination (MMSE) and school attendance was increased, the self-injurious behaviors and suicidal ideations were decreased at follow-up after 1 year (36). A previous review systematically summarized data on the burden associated with SCZ, MDD, BD, and other major mental disorders, revealing that SCZ and BD had the highest disability rating (41). Green et al. reported that individuals with SCZ exhibit impaired social cognition, which manifests as difficulties identifying emotions, feeling connected to others, inferring people's thoughts, and reacting emotionally to others (42). Currently, available studies on the clinical outcomes of ECT in adolescents failed to focus on social function or cognition after ECT in the long term. Our outcomes showed that the PSP score for SCZ patients was lower than MDD and BD, which also indicated some early-onset SCZ adolescents with cognitive deficits. In the present study, there was no correlation between the PSP score and readmission. Whether ECT could improve the social function of adolescents in the long term needs to be further examined.

## Limitations

This retrospective study has a number of limitations. First, we did not record the side effect of ECT and the cognitive impairment of outpatients at follow-up. Previous meta-analyses suggested that cognitive function was impaired immediately following ECT, but returned to at least baseline by two weeks after ECT (43). However, another study reported that autobiographical memory deficits commonly occurred at the completion of the ECT course, and the deficits were long-lasting (44). Second, our results only detected the general increase in the kinds of drugs without recording the exact dose. The changes in dose of oral medication after the ECT course should be assessed by the use of equivalent does. Finally, future independent site validation should be considered, which limits the generalizability of the current study.

## Conclusions

Overall, we provided retrospective evidence suggesting that ECT is an effective treatment for adolescents with severe mental disorders. The benefits of ECT include reducing the risk of relapse and the burden of oral medications. The data presented in this study showed that patients aged 14–18 years with SCZ, BD, and MDD responded well to ECT in the short term, also obtaining long-term benefits. However, whether patients younger than 14 years respond similarly to adolescents and adults remains unclear.

## Data availability statement

The raw data supporting the conclusions of this article will be made available by the authors, without undue reservation.

## Ethics statement

The studies involving human participants were reviewed and approved by the Institutional Review Board at the Affiliated Brain Hospital of Nanjing Medical University. Written informed consent to participate in this study was provided by the participants' legal guardian/next of kin.

## Author contributions

YS and YL designed the study. XZ and JL conducted the literature searches and analysis. QS and CC performed the statistical analysis. FR and GX managed the assessment of the risk bias. QS wrote the manuscript. YL contributed to the final review of the manuscript. All authors contributed to the article and approved the submitted version.

## Funding

This work was supported by the Scientific Research Foundation of Nanjing Medical University (201715048 and ZKX17030).

## Conflict of interest

The authors declare that the research was conducted in the absence of any commercial or financial relationships that could be construed as a potential conflict of interest.

## Publisher's note

All claims expressed in this article are solely those of the authors and do not necessarily represent those of their affiliated organizations, or those of the publisher, the editors and the reviewers. Any product that may be evaluated in this article, or claim that may be made by its manufacturer, is not guaranteed or endorsed by the publisher.
